# Regional disparities of antidementia drug treatment in Germany: what can we learn for the new generation of Alzheimer’s therapies

**DOI:** 10.1186/s13195-025-01902-8

**Published:** 2025-12-04

**Authors:** Moritz Platen, Eva Gläser, Volker Dahling, Daniela Gesell, Michael Hauptmann, Dirk Horenkamp-Sonntag, Daniela Koller, Denise Kubat, Ursula Marschall, Cordula Riederer, Hannah Scheibner, Jennifer Schroth, Enno Swart, Bernhard Michalowsky

**Affiliations:** 1 Research Group for Patient-Reported Outcomes & Health Economics Research, German Center for Neurodegenerative Diseases (DZNE), site Rostock/Greifswald, Ellernholzstrasse 1-2, Greifswald, D-17489 Germany; 2 Research Group Geriatric Psychiatry Research, Brandenburg Medical School Theodor Fontane, Rüdersdorf, Germany; 3 Faculty of Health Science Brandenburg, Brandenburg Medical School Theodor Fontane, Neuruppin, Germany; 4 Center for Mental Health, Immanuel Hospital Rüüdersdorf, Brandenburg Medical School Theodor Fontane, Rüüdersdorf, Germany; 5 Institute of Medical Data Processing, Biometrics and Epidemiology (IBE), Faculty of Medicine, LMU Munich, Munich, Germany; 6 Department of Palliative Medicine, LMU University Hospital, LMU Munich, Munich, Germany; 7 Institute of Biostatistics and Registry Research, Brandenburg Medical School Theodor Fontane, Neuruppin, Germany; 8 Techniker Krankenkasse, Hamburg, Germany; 9 Institute für Social Medicine and Health System research, Medical Faculty, Otto-von- Guericke-University, Magdeburg, Germany; 10 BARMER, Wuppertal, Germany; 11 DAK-Gesundheit, Hamburg, Germany; 12 Department of Health Research Methods, Evidence and Impact, McMaster University, Hamilton, Canada

**Keywords:** Alzheimer’s disease, Antidementia drug treatment, Disease-modifying treatments, geographical variation, spatial analysis, Rural population, Deprivation, Healthcare disparities, Real-world evidence, Real-world data

## Abstract

**Background:**

Current antidementia drugs can temporarily slow cognitive decline in Alzheimer’s disease but are underused. Regional and socioeconomic disparities, including limited specialist access in rural or deprived areas, may exacerbate inequities and challenge the rollout of emerging disease-modifying therapies. This study aimed to evaluate associations between regional contextual factors and antidementia drug prescription (AD-Rx) among newly diagnosed people living with Alzheimer’s disease (PlwAD) in Germany and to identify spatial clustering of prescribing patterns.

**Methods:**

This study analyzed anonymized claims data from three statutory health insurers for 53,753 PlwAD who received their first diagnosis between January 2020 and December 2022. Regions, defined by three-digit postal codes (ZIP3, *n* = 576), were categorized by the German Index of Socioeconomic Deprivation (GISD) quintiles and Degree of Urbanization (urban, suburban, rural). Multilevel logistic regression with random intercepts for ZIP3 was used to assess associations between receiving AD-Rx (dichotomous) and urbanization and deprivation, adjusting for age, sex, the Charlson Comorbidity Index, the long-term care level and the year of diagnosis. Global Moran’s I was used to evaluate large-scale spatial clustering, and regional Moran’s I was calculated to detect regional hotspots and coldspots.

**Results:**

Overall, 64% of PlwAD received at least one AD-Rx. Rural residency was associated with slightly lower odds of receiving AD-Rx compared to urban areas (OR 0.92; 95%CI 0.87–0.98; *p* = 0.010), whereas deprivation was not. Interaction models demonstrated that an increased deprivation further reduced AD-Rx odds in rural areas (OR per GISD unit = 0.98; 95% CI 0.96–0.99; *p* = 0.024). Global Moran’s I revealed no significant large-scale clustering (I = 0.011; *p* = 0.613), but regional analysis identified several regional hotspots (high-high clusters) predominantly in moderately deprived urban areas and coldspots (low-low clusters) in highly deprived or rural areas.

**Conclusion:**

Alzheimer’s patients in rural and high-deprivation regions face limited access to recommended antidementia medications. Targeted interventions, such as teleconsultations, expanding specialist outreach, and collaborative care models in underserved areas, as well as regional dementia networks and national registries, could promote the equitable delivery of current and future Alzheimer’s antibody therapies. However, further qualitative and quantitative research is needed to identify the underlying regional causes of these treatment disparities.

**Trial registration:**

DRKS00031944.

**Supplementary Information:**

The online version contains supplementary material available at 10.1186/s13195-025-01902-8.

## Introduction

Dementia represents a growing global health challenge. In 2019, approximately 57 million people were living with dementia worldwide, a figure projected to exceed 150 million by 2050 [1]. In Germany, estimates predict an increase from 1.8 million people with dementia in 2021 to 3.0 million by 2070 [2, 3]. Although recent birth-cohort studies have indicated a declining age-specific dementia risk, suggesting that earlier projections may have been too high, dementia continues to impose substantial societal and economic burdens [4, 5]. Worldwide costs of dementia now exceed US $1 trillion annually, encompassing medical care, social services, and informal caregiving [6]. On a per-person basis, annual costs for people with dementia are nearly three times higher than for age-matched individuals without dementia [7].

Current pharmacological options (donepezil, galantamine, rivastigmine, and memantine) are approved only for people living with Alzheimer’s disease (PlwAD). These agents can temporarily slow cognitive decline but do not alter the underlying disease trajectory [8]. Multiple studies have highlighted a persistent gap between dementia diagnoses and antidementia drug prescription (AD-Rx), indicating the underuse of guideline-recommended evidence-based pharmacotherapy [9–11]. Use of symptomatic antidementia drugs varies markedly across European countries, with divergent 10-year trends (declines, stability, and increases), underscoring the need for context-specific regional analyses [12]. On the one hand, their benefits for patients are partially questioned [13–15]. On the other hand, one key factor is limited access to specialist care, such as neurologists and psychiatrists, who more frequently detect specific dementia diagnoses and prescribe the majority of antidementia drugs [9]. In contrast, although the prevalence of dementia diagnoses made by general practitioners in Germany remained stable between 2010 and 2021, the rate of AD-Rx prescribed by primary care physicians declined over that period, which entails faster cognitive and functional decline in patients, greater caregiver burden, and downstream economic consequences for the healthcare system [10, 13, 16–18].

Furthermore, evidence suggests that inequalities in dementia care arise from regional and socioeconomic disparities [19, 20]. Patients in rural areas often receive dementia diagnoses later and consult specialists less frequently than those in urban regions [21]. However, studies examining urban-rural differences in AD-Rx have been inconclusive, and it remains unclear whether rurality per se contributes to lower prescription rates [22, 23]. Instead, socioeconomic deprivation may be a stronger determinant of reduced access to antidementia medications [20, 24].

These disparities have significant and critical implications for emerging disease-modifying therapies (DMTs), particularly anti-amyloid antibodies such as donanemab and lecanemab, which are approved by the European Medicines Agency (EMA) or are currently undergoing approval [25, 26]. These novel agents aim to slow the progression of early Alzheimer’s disease but require biomarker confirmation, specialized infusion facilities, and close safety monitoring [27–29]. Health systems could struggle to scale up these resource-intensive services uniformly, potentially exacerbating existing inequities in dementia care.

Only a few prior German studies have assessed urban-rural differences in AD-Rx. These analyses did not find significant urban-rural disparities in the provision of antidementia drugs or partially revealed counterintuitive findings [30–32]. However, they suffer from limited comparability and generalizability, as well as small sample sizes and the lack of consideration of socioeconomic heterogeneity within regions. Moreover, international research has identified supra-regional clusters of AD-Rx that transcend simple urban-rural categorizations [33, 34].

Accordingly, the present study uses nationwide statutory health insurance data to examine (i) whether regional contextual factors, specifically the degree of urbanization and socioeconomic deprivation, are associated with variation in antidementia drug prescriptions of incident PlwAD and (ii) if spatial clustering patterns in antidementia prescribing rates exist. By addressing these questions, the analysis aims to generate new insights for planning equitable dementia treatment in Germany, an urgent priority given the expected rise in demand for both established and disease-modifying drug treatments.

## Methods

### Study design

This analysis utilizes data from the mixed-methods RegioDem study (Regional Variations in Healthcare for People Living with Dementia in Germany), which examines how regional factors influence dementia care [35]. Anonymized claims data were provided by three nationwide statutory health insurers, comprising all continuously insured individuals aged ≥ 18 years with at least one recorded dementia diagnosis between January 2019 and December 2023.

This real-world data included inpatient and outpatient diagnoses (with diagnostic certainty) coded according to the German modification of the 10th edition of the International Classification of Diseases and Related Health Problems (ICD‑10‑GM) [36], all prescribed medications classified by the Anatomic-Therapeutic Chemical (ATC) codes, the German Pharmazentralnummer (PZN; a unique national pharmaceutical identification number), the care level, assigned by the long-term care insurance for the amount of care and support a patient receives owing to their functional impairment, ranging from 1 to 5, with 1 indicating some problems and 5 indicating extreme problems and demographic characteristics (sex, age, region of residence).

The trial was approved by the Ethics Committee of Brandenburg Medical School Theodor Fontane (Ref. 152092023‑BO‑E). A full description of the study design has been published previously [35].

### Study population

Incident patients with an index diagnosis of Alzheimer’s disease (ICD‑10 codes: F00* or G30*) were included in the present analyses if the diagnosis appeared as a confirmed outpatient diagnosis in at least two of four consecutive quarters (so-called M2Q criteria) or as a primary inpatient diagnosis between January 2019 and December 2023. Continuous insurance enrollment for at least 12 months before and after the first diagnosis (index date) was required to ensure complete observation, and individuals aged < 65 years and in the top first percentile of age at diagnosis were excluded to remove extreme outliers. This incident cohort was stratified into those who received at least one prescription of antidementia medication after diagnosis and those who did not, using the ATC code for antidementia drugs (N06D), reflecting real-world prescribing and reimbursement. For sensitivity analyses, a narrower, guideline-based definition limited to cholinesterase inhibitors (N06DA), memantine (N06DX01), and Ginkgo biloba restricted to the standardized extract EGb 761 at a total daily dose of 240 mg (identified via PZN within N06DP01) was used. Table 1 lists all ICD‑10 and ATC codes used.

**Table 1:** Included ICD-10 diagnoses and ATC codes

ICD-10 code	Description
F00	Dementia in Alzheimer disease
F00.0	Dementia in Alzheimer disease with early onset
F00.1	Dementia in Alzheimer disease with late onset
F00.2	Dementia in Alzheimer disease, atypical or mixed type
F00.9	Dementia in Alzheimer disease, unspecified
G30	Alzheimer disease
G30.0	Alzheimer disease with early onset
G30.1	Alzheimer disease with late onset
G30.8	Other Alzheimer disease
G30.9	Alzheimer disease, unspecified

**ATC code**	**Active substance**
N06DA02	Donepezil
N06DA03	Rivastigmin
N06DA04	Galantamin
N06DP01	Ginkgo biloba^a^
N06DX01	Memantin
N06DX13	Nicergolin^b^
N06DX18	Nimodipin^b^

*ATC* Anatomical therapeutic chemical, *ICD-10* International Statistical Classification of Diseases and Related Health Problems, 10th revision

^a^Only ginkgo biloba extract EGb 761 in a daily dose of 240 mg is recommended in the national dementia guideline and was considered using the German national pharmaceutical identifier

^b^Not recommended according to the national dementia guideline

### Regionalization, deprivation and urbanization

The regional unit is based on the first three digits of the German postal code (ZIP3, *n* = 671). These regions were linked to the German Index of Socioeconomic Deprivation (GISD) [37]. This composite index measures the level of socioeconomic deprivation, understood as a lack of material and social resources that constrain living conditions and opportunities for participation, using administrative data on education, employment, and income situations at the district and municipality levels. The index is used to assess the overall average level of deprivation in a given region or population residing in that region, providing a valuable tool for analyzing socioeconomic inequalities in health conditions, diseases, and their determinants at the regional level. The index is demonstrated by quintiles, ranging from the lowest deprivation to low, moderate, high, and highest deprivation. Additionally, the defined regions were linked to the Degree of Urbanization (DEGURBA), operationalized as urban, suburban, and rural [38]. To ensure stable regional estimates and comparability across contextual strata, we retained ZIP3×GISD×DEGURBA regions that contained at least 25 dementia patients. The final sample comprised *N* = 53,753 incident PlwAD in 576 areas with complete data.

### Statistical analyses

Distributions of patient characteristics, including regional urbanization and deprivation indicators, were presented using descriptive statistics. T-tests (for numeric variables) and chi-square test (for categorical variables) were used to test for group differences between PlwAD with and without an AD-Rx. A mixed-effects logistic regression model with random intercepts for ZIP3 regions assessed the association between antidementia prescriptions (dichotomous) and the degree of urbanization (categorical) and deprivation (categorical), reporting odds ratios (OR) adjusted for age at diagnosis (years), comorbidity (Charlson Comorbidity Index (CCI) [39]), gender (dichotomous), the long-term care level and the year of diagnosis to control for the COVID-19 pandemic.

In sensitivity analyses, ordinal scores of GISD were modelled as a continuous variable to assess trends in the likelihood of receiving AD-Rx. Furthermore, the stated mixed-effects logistic regression model was specified to explore the interaction between GISD and DEGURBA, using a categorical interaction model that combined GISD quintiles and urbanization categories (Model 1), as well as a continuous interaction term between GISD and rural residence (Model 2). Multiple testing was controlled using the Holm-Bonferroni method (FWER α = 0.05) over the 14 GISD×DEGURBA contrasts per outcome in Model 1. Finally, a comprehensive interaction model including both predictors and their interaction was evaluated (Model 3).

For spatial analysis, prescription proportions by regions were aggregated, building a 5‑nearest‑neighbor (ZIP3 regions) weight matrix, and calculated global and regional Moran’s I. Regions with significant regional autocorrelation (*p* ≤ 0.05) were flagged, compared to the global mean rate, and classified into the four clusters according to Anselin [40] (Low–Low, Low–High, High–Low, High–High), identifying hotspots, coldspots and spatial outliers. Both the regression models and the spatial analysis were additionally carried out and reported only for guideline-recommended antidementia drug treatment in PlwAD.

All statistical analyses were conducted with STATA/IC software, version 18 [41]. Choropleth maps were constructed to illustrate regional disparities. All cartography was conducted using QGIS software, version 3.40.7 [42].

## Results

### Sample characteristics and distribution in terms of DEGURBA and GISD

A total of 53,753 incident PlwAD were included in the analysis, of whom 36% (*n* = 19,116) did not receive any AD-Rx and 64% (*n* = 34,637) received at least one prescription after diagnosis.

The mean age at diagnosis was 82.9 years, with minimal difference between those without AD-Rx and those with AD-Rx (83.0 vs. 82.8 years; *p* = 0.029). Females comprised 62.9% of the overall sample, with similar proportions across groups (63.5% vs. 62.6%; *p* = 0.031). The mean CCI was 4.0 overall and significantly higher in the untreated group (4.2 vs. 3.9; *p* ≤ 0.001). When stratified by DEGURBA categories, 46.7% of PlwAD resided in cities, 40.8% in towns and suburbs, and 12.5% in rural areas, with no significant AD-Rx differences between groups (*p* = 0.064). Across GISD quintiles, there was a fairly even spread: 19.7% in the least deprived (1st quintile) through 25.1% in the most deprived (5th quintile), with no significant differences by treatment status (*p* = 0.0625). Table 2 summarizes the patient characteristics and the distribution in terms of DEGURBA and GISD.

**Table 2:** Baseline patient characteristics and assigned regional context characteristics

	Total Sample		PlwAD without AD-Rx		PlwAD with AD-Rx		*p*-value
	*n* = 53,753		*n* = 19,116 (36%)		*n* = 34,637 (64%)		
Age at diagnosis, mean (SD)	82.9	(7.6)	83.0	(7.6)	82.8	(7.6)	0.029^b^
Sex (female), n (%)	33,839	(62.9)	12,150	(63.5)	21,689	(62.6)	0.031^c^
CCI, mean (SD)	4.0	(2.7)	4.2	(2.8)	3.9	(3.9)	< 0.001^b^
Care level^a^							
No care level, n (%)	22,122	(41.1)	7,282	(38.0)	14,840	(42.8)	< 0.001^c^
Care level: 1, n (%)	2,661	(4.9)	573	(3.0)	2,088	(6.0)	
2, n (%)	10,146	(18.8)	2,822	(14.7)	7,324	(21.1)	
3, n (%)	12,258	(22.8)	4,731	(24.7)	7,527	(21.7)	
4, n (%)	5,504	(10.2)	3,016	(15.7)	2,488	(7.1)	
5, n (%)	1,062	(1.9)	692	(3.6)	370	(1.0)	
Degree of urbanization (DEGURBA)							
Cities, n (%)	25,135	(46.7)	8,888	(46.5)	16,247	(46.9)	0.064^c^
Towns and suburbs, n (%)	21,964	(40.8)	7,776	(40.6)	14,188	(40.9)	
Rural areas, n (%)	6,654	(12.3)	2,452	(12.8)	4,202	(12.1)	
Socioeconomic deprivation (GISD)							
1 st quintile, n (%)	10,626	(19.7)	3,771	(19.7)	6,855	(19.7)	0.625^c^
2nd quintile, n (%)	8,700	(16.1)	3,035	(15.8)	5,665	(16.3)	
3rd quintile, n (%)	8,028	(14.9)	2,875	(15.0)	5,153	(14.8)	
4th quintile, n (%)	12,893	(23.9)	4,588	(24.0)	8,305	(23.9)	
5th quintile, n (%)	13,506	(25.1)	4,847	(25.3)	8,659	(25.0)	

*AD-Rx* Antidementia drug prescription, *CCI* Charlson Comorbidity Index, *DEGURBA* Degree of urbanisation, *GISD* German Index of Socioeconomic Deprivation, *PlwAD* People living with Alzheimer’s disease

^a^The care level assigned by the long-term care insurance indicate the amount of care and support a patient receives owing to their functional impairment, ranging from 1 to 5, with 1 indicating some problems and 5 indicating extreme problems

^b^Differences in means: *t*-Test two-tailed

^c^Differences in proportions: chi-square tests

### Association between regional contextual factors and prescription of antidementia drugs

Living in rural areas was associated with a 8% reduced chance of receiving AD-Rx compared to living in cities (OR 0.92; 95% CI 0.87–0.98; *p* = 0.010). Additionally, residency in towns and suburbs compared to city dwellings showed no significant differences (OR 0.99; 95% CI 0.95–1.04; *p* = 0.967). Socioeconomic deprivation, modelled categorically by GISD quintiles, was not significantly associated with AD-Rx in any quintile. However, the predicted probabilities show a very slight peak in the second quintile (65.1%; 95% CI 64.1% – 66.1%), are modestly lower in the third quintile (64.1%; 95% CI 63.1% – 65.1%) and fourth quintile (64.4%; 95% CI 63.6% – 65.3%), and lowest in the fifth quintile (64.2%; 95% CI 63.3% – 65.1%) with slight overall variation across groups of only around 1.4% points. Moreover, the random intercept variance at the ZIP3 level indicates low residual heterogeneity between regions (variance = 0.003; 95% CI, 0.001–0.014). Table 3 presents the results of the multilevel regression model, and Supplementary Fig. 1 illustrates the margins plot of predicted probabilities for each GISD quintile.

**Table 3:** Association between regional contextual factors and prescription of antidementia drugs after diagnosis of PlwAD

	Prescription of antidementia drugs after diagnosis of PlwAD		
	OR (SE)	95%CI	*p*-value
AD-Rx (real-world)^a,^^b^			
Fixed effects			
Degree of urbanization (DEGURBA, ref. cities)			
Towns and suburbs	0.99 (0.02)	0.95–1.04	0.967
Rural areas	0.92 (0.02)	0.87–0.98	0.010
Socioeconomic deprivation (GISD, ref. 1 st quintile)			
2nd quintile	1.03 (0.03)	0.97–1.10	0.239
3rd quintile	0.98 (0.03)	0.92–1.05	0.708
4th quintile	1.00 (0.02)	0.94–1.06	0.885
5th quintile	0.99 (0.02)	0.93–1.05	0.863
Intercept	2.20 (0.07)	2.06–2.35	< 0.001
AD-Rx (guideline-recommended)^a,c^			
Fixed effects			
Degree of urbanization (DEGURBA, ref. cities)			
Towns and suburbs	0.99 (0.02)	0.95–1.03	0.853
Rural areas	0.94 (0.02)	0.88–0.99	0.036
Socioeconomic deprivation (GISD, ref. 1 st quintile)			
2nd quintile	1.03 (0.03)	0.98–1.10	0.196
3rd quintile	1.00 (0.03)	0.94–1.06	0.965
4th quintile	1.01 (0.03)	0.95–1.07	0.668
5th quintile	1.00 (0.02)	0.94–1.06	0.831
Intercept	1.85 (0.06)	1.73–1.98	< 0.001

*CI* Confidence interval, *DEGURBA* Degree of urbanisation, *GISD* German Index of Socioeconomic Deprivation, *OR* Odds ratio, *SE* Standard error

^a^Models were adjusted for age, sex comorbidities (cci score), care level and year of diagnosis

^b^Random intercept variance (Zip code 3 digits): 0.003 (0.002) 95% CI 0.001–0.014) Observations = 53,753; Groups (Zip code 3 digits) = 576 AIC = 67,963.62; BIC = 68,123.68

^c^Random intercept variance (Zip code 3 digits): 0.004 (0.002) 95% CI 0.001–0.012) Observations = 53,753; Groups (Zip code 3 digits) = 576 AIC = 69,899.68; BIC = 70,059.74

In sensitivity analyses, a trend model treating GISD as a continuous variable showed no association with deprivation (OR 0.99 per quintile increase; 95% CI 0.98–1.00; *p* = 0.571), whereas the association with rural residence remained significant (OR 0.92; 95% CI 0.87–0.98; *p* = 0.011). Interaction analyses indicated that disadvantages were concentrated in rural regions. In the categorical specification (Model 1), rural × moderate deprivation (OR 0.86, 95% CI 0.76–0.97; *p* = 0.021) and rural × high deprivation (OR 0.87, 95% CI 0.77–0.99; *p* = 0.039) had lower odds relative to urban × lowest deprivation. However, none remained significant after Holm-Bonferroni correction across the 14 contrasts. The continuous specification (Model 2) supported the same pattern, with increasing deprivation associated with lower odds in rural areas (OR per GISD unit = 0.98; 95% CI 0.96–0.99; *p* = 0.024). The overall interaction model (Model 3), considering main and interaction terms, again showed lower odds of receiving AD-Rx for rural residencies (OR 0.92; 95% CI 0.86–0.98; *p* = 0.010). However, Model 3 was accompanied by multicollinearity, making it difficult to determine the independent contributions of the respective regional context factors.

Analyses restricted to guideline-recommended drugs essentially replicated the primary findings: rural residence remained associated with lower odds of AD-Rx (OR 0.94; 95% CI 0.88–0.99; *p* = 0.041), whereas GISD main effects and the categorical GISD×DEGURBA interactions were non-significant after Holm-Bonferroni correction. The only deviation was Model 2: the continuous GISD×rural interaction was not significant for guideline-recommended AD-Rx (OR per GISD unit 0.98; *p* = 0.127), in contrast to the real-world outcome (OR 0.98; *p* = 0.024). Table 4 presents the combined model; the remaining sensitivity analyses are provided in Supplementary Tables 1–3.

**Table 4:** Association between regional contextual factors and prescription of antidementia drugs after diagnosis of plwad: interactions of regional contextual factors

	Interactions (regional contextual factors)		
	OR (SE)	95%CI	*p*-value
AD-Rx (real-world)^a, b^			
Socioeconomic deprivation (GISD, continuous var.)	0.99 (0.01)	0.97–1.01	0.543
Degree of urbanization (DEGURBA, ref. cities)			
Towns and suburbs	1.00 (0.02)	0.96–1.04	0.932
Rural areas	0.92 (0.02)	0.86–0.98	0.010
GISDxDEGURBA (ref. GISD x cities)			
GISD x towns & suburbs	1.00 (0.01)	0.97–1.03	0.878
GISD x rural areas	1.01 (0.02)	0.96–1.05	0.615
Intercept	2.21 (0.05)	2.10–2.32	< 0.001
AD-Rx (guideline-recommended)^a, c^			
Socioeconomic deprivation (GISD, continuous var.)	0.99 (0.01)	0.97–1.01	0.658
Degree of urbanization (DEGURBA, ref. cities)			
Towns and suburbs	0.99 (0.02)	0.95–1.04	0.978
Rural areas	0.93 (0.02)	0.88–0.99	0.039
GISDxDEGURBA (ref. GISD x cities)			
GISD x towns & suburbs	1.00 (0.01)	0.97–1.03	0.726
GISD x rural areas	1.01 (0.02)	0.97–1.05	0.536
Intercept	1.87 (0.04)	1.78–1.97	< 0.001

*CI* Confidence interval, *DEGURBA* Degree of urbanisation, *GISD* German Index of Socioeconomic Deprivation, *OR* Odds ratio, *PlwAD* People living with Alzheimer’s disease, *SE* Standard error

^a^Models were adjusted for age, sex comorbidities (cci score), care level and year of diagnosis

^b^Random intercept variance (Zip code 3 digits): 0.003 (0.002) 95% CI 0.001–0.014) Observations = 53,753; Groups (Zip code 3 digits) = 576 AIC = 67,963.62; BIC = 68,114.79

^c^Random intercept variance (Zip code 3 digits): 0.004 (0.002) 95% CI 0.001–0.013) Observations = 53,753; Groups (Zip code 3 digits) = 576 AIC = 69,899.05; BIC = 70,050.21

### Spatial autocorrelation analysis

Global Moran’s I for the mean AD-Rx rate across ZIP3 regions was 0.011 (expected − 0.002; sd 0.024; Z = 0.505; *p* = 0.613), indicating no significant overall spatial clustering but rather random patterns of prescription proportions. Local indicators of spatial association identified 17 spatial outlier areas (mean AD-Rx rate = 0.66), mainly in moderately deprived (mean GISD quintile = 2.89) and rural regions (mean DEGURBA = 1.80), comprising both low-high and high-low outliers. Eleven hotspots (high-high clusters; mean AD-Rx rate = 0.77) were found predominantly in urbanized regions (mean DEGURBA = 1.73) with moderate deprivation (mean GISD quintile = 3.36), while 10 coldspots (low-low clusters; mean AD-Rx rate = 0.53) were located in more deprived (mean GISD quintile = 3.94) and rural areas (mean DEGURBA = 2.05). These local patterns suggest regional disparities in antidementia prescribing that are not captured by global measures.

**Fig. 1:**
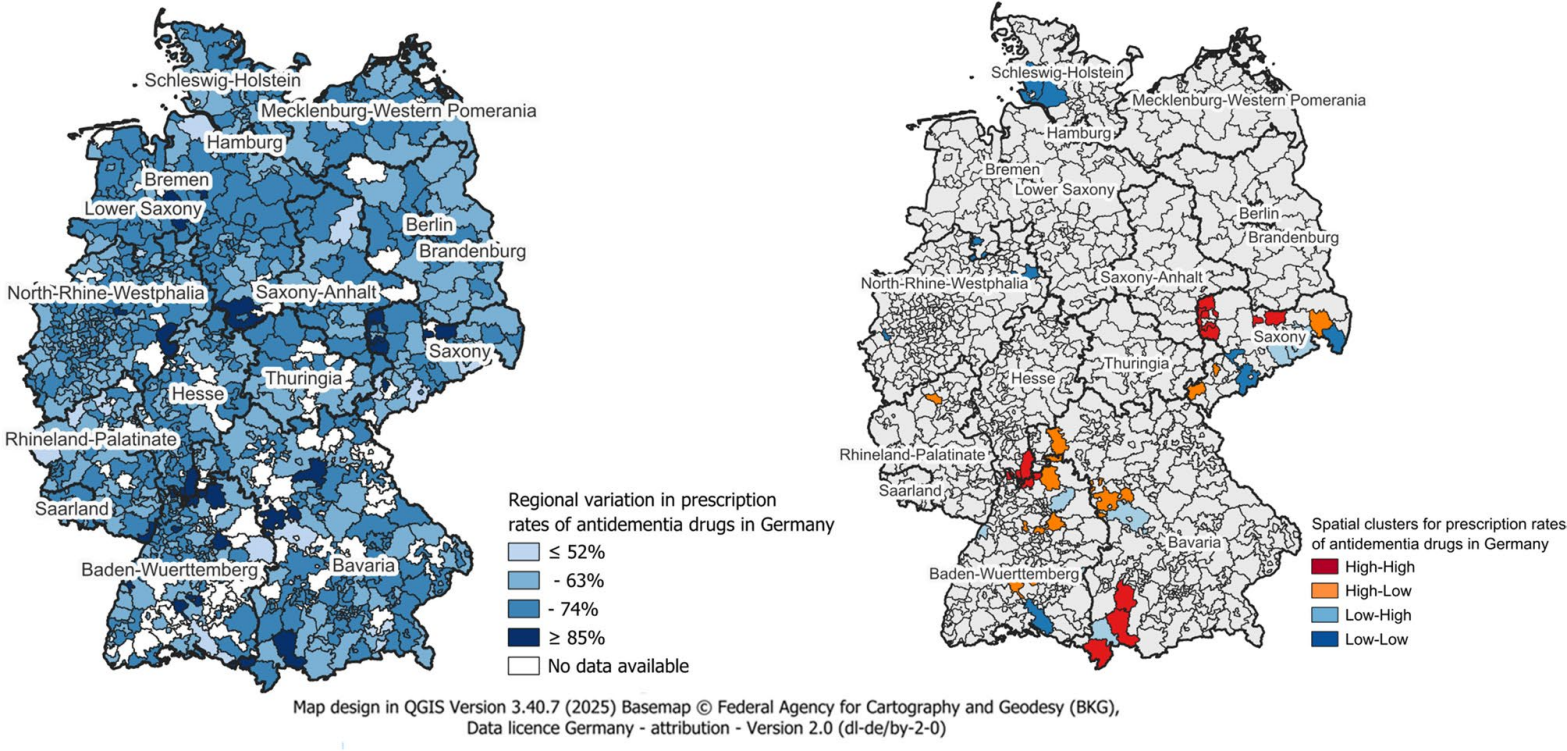
Regional variation and spatial clusters of antidementia prescriptions in Germany

The spatial autocorrelation analysis based on guideline-recommended drugs closely mirrored the primary results: no evidence of large-scale clustering, and a similar regional pattern with hotspots in more urban and moderately deprived areas and coldspots in more suburban and rural areas with higher deprivation. Overall, the spatial result was consistent, corroborating regional disparities seen in the real-world outcome analysis. Tables 5 and 6 reports the results of the spatial autocorrelation analysis. Figure 1 illustrates the regional variation and spatial clusters of real-world antidementia prescriptions in Germany.

**Table 5:** Spatial autocorrelation analysis for real-world AD-Rx

Zip code 3 digits	Moran’s I (local)	*p*-value	AD-Rx, mean (Regions’s vs. global^1^)		DEGURBA, mean	GISD, mean
Hotspots (high AD-Rx mean surrounded by high neighbors)						
010	0.924	0.037	0.78	(> mean)	1.00	2.00
015	1.944	≤ 0.001	0.84	(> mean)	2.00	4.00
041	0.922	0.038	0.71	(> mean)	1.00	4.00
044	1.201	0.007	0.75	(> mean)	1.59	4.31
045	1.061	0.017	0.75	(> mean)	2.42	4.52
477	0.897	0.043	0.78	(> mean)	1.00	5.00
647	1.487	≤ 0.001	0.76	(> mean)	2.00	5.00
694	1.169	0.008	0.75	(> mean)	2.00	1.25
868	0.945	0.033	0.72	(> mean)	2.00	1.39
875	1.677	≤ 0.001	0.72	(> mean)	2.00	1.45
876	0.884	0.046	0.86	(> mean)	2.00	4.00
	Total mean:		**0.77**		**1**,**73**	**3**,**36**
Coldspot (low AD-Rx mean surrounded by low neighbors)						
027	1.452	≤ 0.001	0.54	(< mean)	2.38	5.00
092	1.280	0.004	0.56	(< mean)	2.00	4.03
094	1.532	≤ 0.001	0.50	(< mean)	3.00	4.43
255	0.872	0.049	0.58	(< mean)	2.33	4.89
257	1.404	0.002	0.54	(< mean)	2.72	5.00
328	0.984	0.027	0.51	(< mean)	2.00	4.00
411	1.086	0.014	0.52	(< mean)	1.00	5.00
490	0.967	0.029	0.49	(< mean)	1.00	3.00
492	1.010	0.023	0.54	(< mean)	2.00	3.00
886	1.908	≤ 0.001	0.49	(< mean)	2.00	1.00
	Total mean:		0.53		2.04	3,94
Spatial outliers (low (or high) AD-Rx mean surrounded by high (or low) neighbors						
017	−1.208	0.007	0.57	(< mean)	2.00	4.00
018	−3.143	≤ 0.001	0.46	(< mean)	2.00	4.04
026	−1.911	≤ 0.001	0.72	(> mean)	2.00	4.00
080	−1.774	≤ 0.001	0.76	(> mean)	1.00	4.00
085	−1.225	0.006	0.72	(> mean)	1.00	5.00
566	−1.984	≤ 0.001	0.71	(> mean)	2.00	4.00
715	−1.027	0.021	0.80	(> mean)	2.00	1.90
716	−0.972	0.029	0.72	(> mean)	1.39	1.00
723	−1.805	≤ 0.001	0.78	(> mean)	2.00	3.00
746	−0.897	0.044	0.55	(< mean)	2.00	2.00
747	−2.580	≤ 0.001	0.82	(> mean)	2.00	4.00
764	−0.943	0.034	0.53	(< mean)	2.00	2.00
874	−1.662	≤ 0.001	0.58	(< mean)	1.92	2.08
890	−0.989	0.026	0.53	(< mean)	1.00	1.00
915	−1.319	0.003	0.75	(> mean)	2.00	3.00
917	−1.175	0.008	0.52	(< mean)	2.00	3.00
978	−1.001	0.025	0.70	(> mean)	2.36	1.18
	**Total mean**:		**0.66**		**1**,**80**	**2**,**89**

Moran’s I (global): 0.011 (E [I] = − 0.002; sd [I] = 0.024), Z = 0.505, p = 0.613, ^1^AD-Rx mean (global): 0.64

*AD-Rx* Antidementia drug prescription, *DEGURBA* Degree of urbanisation, *GISD* German Index of Socioeconomic Deprivation

**Table 6:** Spatial autocorrelation analysis for guideline-recommended AD-Rx

**Zip code 3 digits**	**Moran’s I (local)**				***p*** **-value**		**AD-Rx**,**mean(Regions’s vs. global** ^**1**^ **)**				**DEGURBA**,** mean**	**GISD**,** mean**
Hotspots (high AD-Rx mean surrounded by high neighbors)												
010	0.992				0.025		0.74		(> mean)		1.00	2.00
013	0.879				0.048		0.70		(> mean)		1.00	2.00
015	1.993				≤ 0.001		0.84		(> mean)		2.00	4.00
273	0.927				0.037		0.73		(> mean)		3.00	4.47
373	1.048				0.018		0.67		(> mean)		3.00	4.16
592	0.954				0.032		0.67		(> mean)		2.00	3.63
593	0.933				0.035		0.67		(> mean)		2.00	3.62
599	0.873				0.049		0.76		(> mean)		2.00	3.00
694	0.980				0.027		0.71		(> mean)		2.00	1.24
776	1.005				0.024		0.76		(> mean)		1.00	3.00
999	1.012				0.023		0.74		(> mean)		2.00	5.00
	Total mean:						0.73				1.91	3.28
Coldspot (low AD-Rx mean surrounded by low neighbors)												
027	1.854				≤ 0.001		0.47		(< mean)		2.38	5.00
079	1.391				0.002		0.48		(< mean)		2.00	5.00
255	1.126				0.011		0.54		(< mean)		2.32	4.88
257	1.216				0.006		0.52		(< mean)		2.72	5.00
411	1.707				≤ 0.001		0.45		(< mean)		1.00	5.00
417	1.185				0.008		0.52		(< mean)		2.00	4.00
492	0.903				0.042		0.50		(< mean)		2.00	3.00
533	1.034				0.02		0.55		(< mean)		2.00	2.52
535	0.963				0.03		0.37		(< mean)		2.00	4.18
886	1.864				≤ 0.001		0.44		(< mean)		2.00	1.00
	Total mean:						0.48				2.04	3.96
Spatial outliers (low (or high) AD-Rx mean surrounded by high (or low) neighbors												
018	−2.545		≤ 0.001				0.46		(< mean)		2.00	4.03
042	−1.112		0.013				0.54		(< mean)		1.00	4.00
080	−1.048		0.019				0.71		(> mean)		1.00	4.00
396	−1.037		0.020				0.70		(> mean)		3.00	5.00
566	−1.688		≤ 0.001				0.67		(> mean)		2.00	4.00
576	−1.247		0.005				0.68		(> mean)		3.00	4.44
682	−1.026		0.021				0.47		(< mean)		1.00	3.00
683	−1.083		0.015				0.72		(> mean)		1.00	3.00
723	−1.222		0.006				0.77		(> mean)		2.00	3.00
747	−1.246		0.005				0.71		(> mean)		2.00	4.00
764	−0.880		0.048				0.48		(< mean)		2.00	2.00
784	−1.120		0.012				0.66		(> mean)		1.00	3.00
833	−1.211		0.007				0.68		(> mean)		3.00	1.46
837	−0.958		0.031				0.70		(> mean)		2.00	1.00
874	−1.685		≤ 0.001				0.54		(< mean)		1.91	2.08
890	−1.195		0.007				0.50		(< mean)		1.00	1.00
915	−1.638		≤ 0.001				0.73		(> mean)		2.00	3.00
917	−1.247		0.005				0.48		(< mean)		2.00	3.00
978	−1.573		≤ 0.001				0.70		(> mean)		2.36	1.18
	Total mean:						0.63				1.86	2.96

*AD-Rx* Antidementia drug prescription, *DEGURBA* Degree of urbanisation, *GISD* German Index of Socioeconomic Deprivation

Moran’s I (global): 0.009 (E[I] = − 0.002; sd[I] = 0.024), Z = 0.431, *p* = 0.666, ^1^AD-Rx mean (global): 0.61

## Discussion

This study provides valuable evidence regarding the association between the supply of antidementia drugs and regional context factors, such as the degree of urbanization and socioeconomic deprivation, indicating statistically significant disadvantages in AD-Rx treatment for incident PlwAD residing in rural regions. In contrast, socioeconomic deprivation showed no overall main effect. Spatial autocorrelation analyses revealed no large-scale clustering, but regional hot- and coldspots that mark regions with systematically higher or lower prescription rates, which are likely related to both the level of urbanization and deprivation.

In a recent systematic review, Arsenault-Lapierre et al. [22] found no clear evidence of urban-rural differences in Alzheimer’s drug prescriptions. The present results refine this picture and add nuance by indicating a slight but consistent rural disadvantage in AD-Rx compared to urban areas among incident PlwAD after adjustment for demographics, comorbidity, care level, and year of diagnosis. One reason could be a delayed diagnosis: rural patients consult neurologists or psychiatrists, who perform the neuropsychological tests needed to initiate treatment, far less often. Instead, they are more frequently managed by primary care physicians, whose AD-Rx rate decreased between 2010 and 2021 in Germany [10, 21]. However, once PlwAD access specialized care in rural areas, the likelihood of receiving AD-Rx increases. A German study even found that neurologists in rural areas prescribe more antidementia drugs per capita than their counterparts in urban areas, underscoring the benefit of specialist availability [30]. Even if the individual-level association is small, average gaps can accumulate into population-level consequences in an aging population. Given the challenges in rolling out DMTs and the accelerated aging of rural populations, memory clinics and specialist outreach in these areas are especially important to ensure timely diagnosis and evidence-based treatment, as recommended by dementia-specific guidelines [43]. Expanding teleconsultation with neurologists and psychiatrists, initiated by general practitioners, also appears feasible within the healthcare system and can partly offset the limited specialist availability for PlwAD in rural areas [44, 45].

Most earlier studies consistently report that higher socioeconomic deprivation reduces the likelihood of receiving AD-Rx [20, 23, 24]. In contrast, after adjustment, the present analysis suggests no overall main effect of deprivation (by GISD quintile) at the regional level on AD-Rx. Interaction analyses revealed that, specifically in rural regions, higher deprivation could be associated with modestly lower odds of AD-Rx prescription. However, this association did not remain significant after Holm-Bonferroni correction and was not statistically significant for the guideline-recommended outcome, although the point estimates were directionally similar. This nuanced pattern suggests that in contexts where healthcare infrastructure is already limited, additional socioeconomic disadvantages, such as low educational levels and limited social support, may further exacerbate barriers to treatment, even though the evidence in our data is not definitive. It is essential to note that nearly 50% of the German population resides in regions with high or the highest deprivation, underscoring the importance of the findings of this study. Further research is needed to obtain reliable findings and conclusions.

In light of an underserved situation in more deprived, rural regions, primary care-based care models such as collaborative dementia care management appear particularly suitable in such areas to address access gaps for guideline-based dementia treatment and care. This holistic approach, which addresses diagnostic, medical, pharmaceutical, and social needs, has already been proven safe and cost-effective, resulting in an increase in AD-Rx rates from 29% to 39% within six months after the intervention [46–48]. However, disparities are likely to widen under DMTs given the specific diagnostic and monitoring requirements. Recent modelling of Germany’s biomarker-based diagnostic capacity projects substantial bottlenecks upon DMT introduction, with long waits for specialist assessments [49]. Regarding the new generation of Alzheimer’s therapies and their related logistical, monitoring, and access challenges, further research is urgently needed to evaluate whether integrated collaborative care models could improve access to DMTs and, thus, patient-relevant outcomes even more. Nevertheless, because deprivation encompasses broader social conditions (such as education, employment, and income), closing treatment gaps will require additional preventive approaches that extend beyond improving medical access.

In the United States, researchers identified spatial clusters of high AD-Rx rates in regions with a high concentration of specialists, high healthcare utilization, and high per capita costs, predominantly located in urban, less deprived areas [33]. Similarly, a Brazilian analysis found AD-Rx hotspots in highly educated, physician-dense regions, while coldspots appeared in structurally weak areas [34]. In contrast, the present global Moran’s I did not show significant clustering of prescribing rates at a large scale, indicating that differences in access to antidementia drugs are relatively scattered rather than concentrated in extensive contiguous regions. Nonetheless, the present study identifies regional hotspots and coldspots that are consistent across both real-world and guideline-recommended prescription practices, with most coldspots occurring more frequently in suburban or rural areas with higher deprivation. These regional variations may reflect local healthcare conditions such as the presence or absence of memory clinics, rural outreach or support programs, or local prescribing behaviour. To address these disparities, policymakers should target regions identified as coldspot areas by pooling resources with neighbouring regions. Establishing regional dementia networks could provide the framework for a more equitable supply chain of antidementia treatments. For instance, Köhler et al. [50] demonstrated that interdisciplinary regional networks resulted in increased AD-Rx compared to usual care, highlighting the potential to allocate patients to their unmet medication needs. However, claims data alone do not capture reasons for non-prescription. Targeted qualitative work in identified hotspots and coldspots is needed to distinguish intentional non-prescription from access deficiencies.

### Limitations

Although this study provides crucial insights, several limitations must be acknowledged. The results are based on claims data, which depend on coding practices and guidelines, reimbursement incentives, budgetary restrictions, and changes in the health and documentation systems. This dependence introduces bias and limits the generalizability of the findings, particularly when compared with other study designs, such as cohort studies. Particularly, the restriction to specific incident Alzheimer’s diagnoses does not correspond to the actual diagnosis and prescribing behaviour, and leads to an underestimation of the actual cases. The present analysis lacked social variables at the individual level (e.g., marital status) that are associated with the detection of dementia and the use of care services, which may lead to residual confounding factors and distort estimates of regional differences [51]. Moreover, prescription data capture dispensing, but not actual prescribing behaviour or medication consumption, which can likewise differ between regions. Reliance on three-digit postal codes may mask heterogeneity at finer spatial scales. Furthermore, the GISD and DEGURBA indices are area-level proxies and cannot fully account for individual socioeconomic status or care preferences, indicating a further need for qualitative research, particularly to gain insights into the rationale behind non-prescribing. Although our incident cohort of 53,753 Alzheimer’s patients is large overall, the imbalance between participants from urban (*n* = 25,135) and rural (*n* = 6,654) regions, together with the small observed effect (OR = 0.92), yields a post-hoc power of 83.03% to detect this difference. To reach the 90% power under these assumptions would require a balanced sample of approximately 13,040 patients per group. Since our data originate from real-world insurance datasets, a larger or balanced sample was not available. This limitation should be taken into account when interpreting the results. Finally, unmeasured factors, such as physician density, provider attitudes, patient cognitive status or dementia severity at the time of treatment initiation, could have influenced the results. While models adjust for index year, within-year pandemic heterogeneity may remain and cannot be fully disentangled. Despite these limitations, this study highlights critical targets for enhancing access to Alzheimer’s disease medications. Future research should focus on collecting more granular spatial data and measuring individual socioeconomic status to refine these insights. Additionally, creating a nationwide dementia registry would allow long-term monitoring and inform policymakers, researchers, and clinicians [52].

## Conclusion

This study revealed that incident cases of people living with Alzheimer’s disease in rural areas are less likely to receive antidementia medications than their urban counterparts, a disparity intensified by higher regional socioeconomic deprivation. Although overall average deprivation did not independently predict prescriptions after adjustment, it still reduced treatment odds in resource-limited rural settings. Local coldspots of prescribing highlight geographic inequalities that warrant further inquiry into interventions, such as expanding memory clinics and specialist outreach, remote specialist consultations, and collaborative care models in high-deprivation rural regions. Establishing regional dementia networks and a nationwide registry with more detailed spatial and socioeconomic data will be crucial for monitoring access, informing policy, and ensuring the equitable delivery of existing and future Alzheimer’s therapies.

## Supplementary Information

Supplementary Material 1. Supplementary Table 1: Association between regional contextual factors and prescription of antidementia drugs after diagnosis of PlwAD: Trend analysis (socioeconomic deprivation). Supplementary Table 2A: Association between regional contextual factors and prescription of antidementia drugs after diagnosis of PlwAD: Interactions of regional contextual factors. Supplementary Table 2B: Association between regional contextual factors and prescription of antidementia drugs after diagnosis of PlwAD: Interactions of regional contextual factors.

## Data Availability

The datasets analyzed during the current study are not publicly available due to data protection reasons and contractual regulations with the data owner.

## Abbreviations

AD-Rx: Antidementia drug prescription
ATC code: Anatomical Therapeutic Chemical code
CCI: Charlson Comorbidity Index
CI: Confidence interval
DEGURBA: Degree of Urbanization
DMTs: Disease-modifying therapies
EMA: European Medicines Agency
GISD: German Index of Socioeconomic Deprivation
ICD 10 GM: German modification of the 10th edition of the International Classification of Diseases and Related Health Problems
OR: Odds ratios
PlwAD: People living with Alzheimer's disease
RegioDem: Regional Variations in Healthcare for People Living with Dementia in Germany
ZIP3: Three digits of the German postal code
