# Supplementary material for: Regional disparities of antidementia drug treatment in Germany: what can we learn for the new generation of Alzheimer’s therapies
Source: Alzheimers Res Ther. 2025 Dec 4;17:259. doi: 10.1186/s13195-025-01902-8 (PMC12696915; doi:10.1186/s13195-025-01902-8)

Supplementary Table 1: Association between regional contextual factors and prescription of antidementia drugs after diagnosis of PlwAD: Trend analysis (socioeconomic deprivation)

	**Prescription of antidementia drugs after diagnosis of PlwAD Trend analysis (socioeconomic deprivation)**		
	**OR (SE)**	**95%CI**	***p*-value**
**AD-Rx (real-world)**			
Fixed effects[a]			
Degree of urbanization (DEGURBA, ref. cities)			
Towns and suburbs	1.00 (0.02)	0.96 – 1.04	0.939
Rural areas	**0.92 (0.02)**	**0.87** – **0.98**	**0.011**
Socioeconomic deprivation (GISD, continuous var.)	0.99 (0.01)	0.98 – 1.00	0.571
Intercept	**2.24 (0.07)**	**2.09 – 2.39**	**<0.001**
**AD-Rx (guideline-recommended)**			
Fixed effects[a]			
Degree of urbanization (DEGURBA, ref. cities)			
Towns and suburbs	0.99 (0.02)	0.95 – 1.04	0.963
Rural areas	**0.94 (0.02)**	**0.88** – **0.99**	**0.041**
Socioeconomic deprivation (GISD, continuous var.)	0.99 (0.01)	0.98 – 1.01	0.890
Intercept	**1.88 (0.06)**	**1.75 – 2.01**	**<0.001**

Abbreviations: CI, confidence interval; DEGURBA, degree of urbanisation ; GISD, German Index of Socioeconomic Deprivation; OR, Odds ratio; PlwAD, people living with Alzheimer's disease; SE, standard error

[a]Models were adjusted for age, sex and comorbidities (cci score).

Random intercept variance (Zip code 3 digits): 0.003 (0.002) 95 % CI 0.001 – 0.014)
Observations = 53,753; Groups (Zip code 3 digits) = 576
AIC = 67,959.88 ; BIC = 68,093.26
Random intercept variance (Zip code 3 digits): 0.003 (0.002) 95 % CI 0.001 – 0.013)
Observations = 53,753; Groups (Zip code 3 digits) = 576
AIC = 69,895.48; BIC = 70,028.86

Supplementary Table 2A: Association between regional contextual factors and prescription of antidementia drugs after diagnosis of PlwAD: Interactions of regional contextual factors

	Interactions (regional contextual factors)

Interactions (regional contextual factors)

	OR (SE)	95%CI	*p*-value (unadjusted)	p-value (Holm)
**AD-Rx (real-world)**				
GISD**x**DEGURBA (ref. 1st quintile **x** cities)				
1st quintile **x** towns & suburbs	0.96 (0.04)	0.88 – 1.04	0.377	1.000
1st quintile **x** rural areas	0.94 (0.08)	0.78 – 1.13	0.541	1.000
2nd quintile **x** cities	1.00 (0.05)	0.91 – 1.11	0.867	1.000
2nd quintile **x** towns & suburbs	1.03 (0.04)	0.95 – 1.12	0.401	1.000
2nd quintile **x** rural areas	0.94 (0.06)	0.81 – 1.08	0.422	1.000
3rd quintile **x** cities	0.95 (0.05)	0.85 – 1.06	0.393	1.000
3rd quintile **x** towns & suburbs	0.99 (0.04)	0.92 – 1.08	0.999	1.000
3rd quintile **x** rural areas	**0.86 (0.05)**	**0.76 – 0.97**	**0.021**	0.294
4th quintile **x** cities	1.01 (0.04)	0.93 – 1.09	0.715	1.000
4th quintile **x** towns & suburbs	0.97 (0.04)	0.89 – 1.06	0.614	1.000
4th quintile **x** rural areas	**0.87 (0.05)**	**0.77 – 0.99**	**0.039**	0.507
5th quintile **x** cities	0.96 (0.03)	0.89 – 1.04	0.382	1.000
5th quintile **x** towns & suburbs	0.98 (0.04)	0.89 – 1.07	0.686	1.000
5th quintile **x** rural areas	0.94 (0.04)	0.85 – 1.04	0.304	1.000
Intercept	2.23 (0.08)	2.07 – 2.40	**<0.001**	
**AD-Rx (guideline-recommended)**				
GISD**x**DEGURBA (ref. 1st quintile **x** cities)				
1st quintile **x** towns & suburbs	0.94 (0.04)	0.86 – 1.02	0.181	1.000
1st quintile **x** rural areas	0.96 (0.08)	0.80 – 1.15	0.671	1.000
2nd quintile **x** cities	1.00 (0.04)	0.90 – 1.10	0.985	1.000
2nd quintile **x** towns & suburbs	1.02 (0.04)	0.95 – 1.11	0.480	1.000
2nd quintile **x** rural areas	0.95 (0.07)	0.82 – 1.10	0.547	1.000
3rd quintile **x** cities	0.95 (0.05)	0.85 – 1.06	0.382	1.000
3rd quintile **x** towns & suburbs	1.00 (0.04)	0.92 – 1.09	0.841	1.000
3rd quintile **x** rural areas	**0.88** (**0.05)**	**0.78** – **0.99**	**0.042**	0.546
4th quintile **x** cities	1.01 (0.04)	0.93 – 1.10	0.716	1.000
4th quintile **x** towns & suburbs	0.98 (0.04)	0.90 – 1.07	0.792	1.000
4th quintile **x** rural areas	**0.87** (**0.05)**	**0.77** – **0.98**	**0.024**	0.336
5th quintile **x** cities	0.97 (0.04)	0.89 – 1.05	0.466	1.000
5th quintile **x** towns & suburbs	0.97 (0.04)	0.89 – 1.07	0.622	1.000
5th quintile **x** rural areas	0.98 (0.04)	0.89 – 1.08	0.717	1.000
Intercept	1.89 (0.07)	1.75 – 2.04	**<0.001**	

Abbreviations: CI, confidence interval; DEGURBA, degree of urbanisation ; GISD, German Index of Socioeconomic Deprivation; OR, Odds ratio; SE, standard error
[a]Models were adjusted for age, sex comorbidities (cci score), care level and year of diagnosis.
[b]Random intercept variance (Zip code 3 digits): 0.003 (0.002) 95 % CI 0.001 – 0.014)
Observations = 53,753; Groups (Zip code 3 digits) = 576
AIC = 67,974.19; BIC = 68,205.39
[c]Random intercept variance (Zip code 3 digits): 0.004 (0.002) 95 % CI 0.001 – 0.013)
Observations = 53,753; Groups (Zip code 3 digits) = 576
AIC = 69,907.25; BIC = 70,138.45
[e]GISD was considered as continuous (mean centered) variable due to multicollinearity

Supplementary Table 2B: Association between regional contextual factors and prescription of antidementia drugs after diagnosis of PlwAD: Interactions of regional contextual factors

Model 2[a,c]	OR (SE)	95%CI	*p*-value
**AD-Rx (real-world)**			
GISDxDEGURBA			
GISD **x** cities	0.99 (0.01)	0.98 – 1.01	0.689
GISD **x** towns & suburbs	0.99 (0.01)	0.98 – 1.01	0.802
GISD **x** rural areas	**0.98 (0.01)**	**0.96 – 0.99**	**0.024**
Intercept	**2.22** (**0.07)**	**2.08** – **2.37**	**<0.001**
**AD-Rx (guideline-recommended)**			
GISDxDEGURBA			
GISD **x** cities	0.99 (0.01)	0.98 – 1.01	0.962
GISD **x** towns & suburbs	1.00 (0.01)	0.98 – 1.01	0.957
GISD **x** rural areas	0.98 (0.01)	0.97 – 1.00	0.127
Intercept	**1.87** (**0.06)**	**1.75** – **1.99**	**<0.001**

Abbreviations: CI, confidence interval; DEGURBA, degree of urbanisation ; GISD, German Index of Socioeconomic Deprivation; OR, Odds ratio; PlwAD, people living with Alzheimer's disease; SE, standard error

[a]Models were adjusted for age, sex and comorbidities (cci score).

Random intercept variance (Zip code 3 digits): 0.003 (0.002) 95 % CI 0.001 – 0.014)
Observations = 53,753; Groups (Zip code 3 digits) = 576
AIC = 67,961.51 ; BIC = 68,094.89
Random intercept variance (Zip code 3 digits): 0.004 (0.002) 95 % CI 0.001 – 0.013)
Observations = 53,753; Groups (Zip code 3 digits) = 576
AIC = 69,896.68; BIC = 70,030.06

Supplementary Figure1

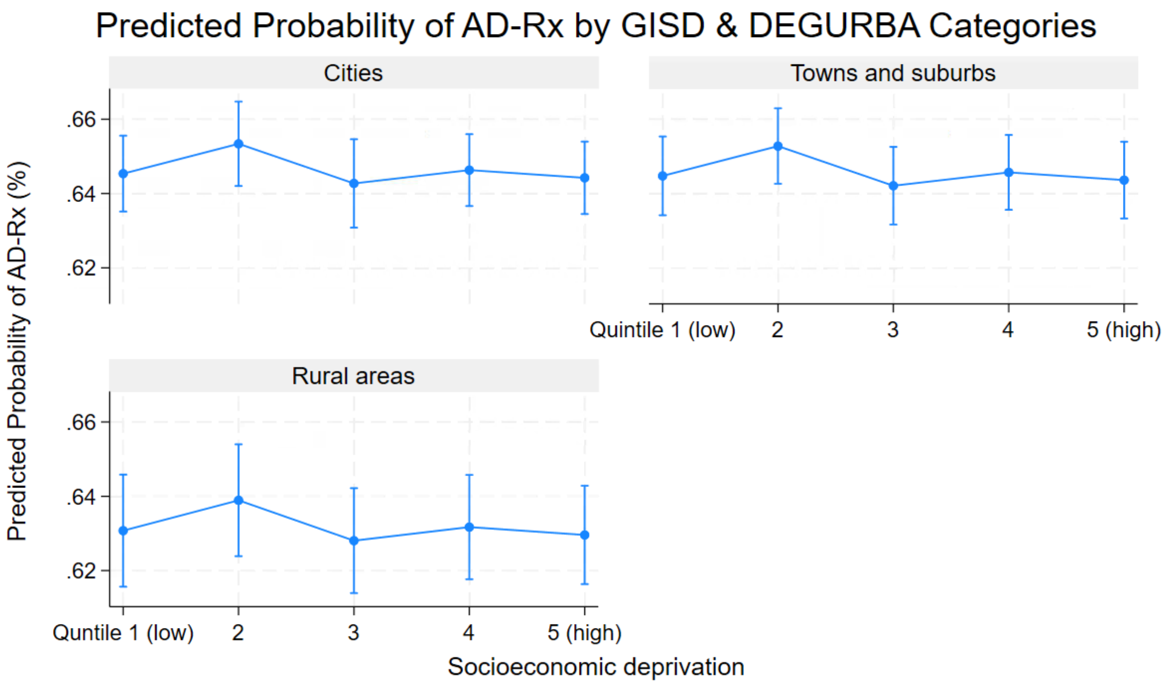

Supplement: Supplementary file 1 — Supplementary Material 1. Supplementary Table 1: Association between regional contextual factors and prescription of antidementia drugs after diagnosis of PlwAD: Trend analysis (socioeconomic deprivation). Supplementary Table 2A: Association between regional contextual factors and prescription of antidementia drugs after diagnosis of PlwAD: Interactions of regional contextual factors. Supplementary Table 2B: Association between regional contextual factors and prescription of antidementia drugs after diagnosis of PlwAD: Interactions of regional contextual factors. [file 13195_2025_1902_MOESM1_ESM.pdf]
